# Effectiveness of Apple Cider Vinegar and Mechanical Removal on Dental Plaque and Gingival Inflammation of Children With Cerebral Palsy

**DOI:** 10.7759/cureus.26874

**Published:** 2022-07-15

**Authors:** Nour Asaad, Mohannad Laflouf

**Affiliations:** 1 Department of Pediatric Dentistry, Damascus University, Faculty of Dentistry, Damascus, SYR

**Keywords:** mechanical removal, cerebral palsy, manual brushing, dental plaque, apple cider vinegar

## Abstract

Background

This study was designed to evaluate the effect of apple cider vinegar (ACV) 5% and mechanical plaque removal with a manual toothbrush on dental plaque and gingivitis. The objective was to study available, natural, and inexpensive ways to improve oral health status among the studied group.

Materials and methods

A randomized clinical trial was performed on 50 children with cerebral palsy aged from three to six years. Participants were divided into two groups (n=25, apple cider vinegar, manual brushing without toothpaste). Plaque accumulation and gingival status were evaluated using Turesky of the Quigley-Hein plaque index (TQHPI) and modified gingival index (MGI) seven times: pre-intervention baseline (T0), post-intervention at one month (T1), two months (T2), three months (T3), four months (T4), five months (T5), six months (T6).

Results

Plaque accumulation and gingivitis decreased significantly for the ACV group between T0 and T6 (p<0.05) and demonstrated significantly lower plaque accumulation and gingivitis compared to the manual brushing group (p<0.05) at T5 and T6. The manual brushing group showed decreasing in TQHPI between T0 and T3, then TQHPI increased significantly (p<0.05) at T4, T5, and T6.

Conclusions

To sum up, this in vitro study has demonstrated the possibility of using apple cider vinegar to reduce plaque and gingivitis. In addition, without additives, apple cider vinegar has both mechanical and chemical effects on dental plaque, and it may be a natural, available, inexpensive, and harmless substance that can improve the quality of oral care for difficult groups of children and people with special needs. Unlike toothbrushes, especially electric toothbrushes, they are effective, but they may be expensive and not available to all children.

## Introduction

Maintaining adequate oral care is just one of many important aspects of overall health care. But it can be a challenge when caring for children with special care needs [1], especially children with cerebral palsy (CP) who face many physical challenges throughout their lifetime [2].

Severe motor incoordination in children with CP affects the ability to perform adequate oral hygiene, and cognitive deficits make cooperation for effective oral care more difficult, which is considered to be the main cause of tooth decay and gingivitis [3]. Moreover, dysfunction in the coordination of swallowing mechanisms, drooling, bruxism, and mouth breathing, make it harder to maintain good oral hygiene than in healthy children and others with special needs [4]. Also, some children with cerebral palsy have sensory difficulties or difficulties in swallowing and chewing that prevent them from using toothpaste with brushing. Therefore, combined mechanical and chemotherapeutic measure is highly recommended to maintain the oral hygiene and gingival health of these children with special needs [5]. In such cases, a powered toothbrush is highly recommended. These toothbrushes can be used more easily and efficiently by parents or nurses when the patient is unable to do so, but different powered toothbrushes are considered expensive, especially in poor countries, and may not be available [6,7].

Many studies have shown that mechanical removal procedures without any chemical factor were effective in reducing dental plaque and gingivitis [8-10]. But because of limited access to interdental and gingival areas by using only dental brushes, especially in young children and children with special needs [11], chemical agents, such as chlorhexidine, have been used as an adjunct factor for better plaque and gingivitis control [9]. Due to the limited use of chlorhexidine for young children under twelve and its long-term negative effects [12], studies have been done on natural herbal alternative products that decrease plaque accumulation and gingivitis without chlorhexidine side effects [13].

Apple cider vinegar (ACV) is a fermented apple juice that is composed mainly of acetic acid (4% to 8%) and derives its other compounds (enzymes, fibers, minerals, and vitamins) from the source [14]. ACV contains vitamins B1, B2, and B6, biotin, folic acid, niacin, pantothenic acid, vitamin C and small amounts of sodium (3 mg/ml), phosphorus, potassium (125 mg/ml), calcium (1.5 mg/100ml), iron (0.02 mg/100ml) and magnesium (2.45 mg/100ml) [15]. Studies have shown that acetic acid has a microbial effect against *Streptococcus mutans *and *C. Albicans* [16]. Yagnik et al. investigated the antimicrobial capacity of ACV against *E. coli*, *S. aureus*, and *C. Albicans *and found that the minimum dilution of ACV required for growth inhibition varied for each microbial species. For *C. Albicans*, a 1/2 dilution ACV had the strongest effect; for *S. aureus*, a 1/25 dilution was required, whereas 1/50 ACV dilution was required for *E. coli* [17]. Many studies have also demonstrated the ability of apple cider vinegar to solve dental plaque and reduce its accumulation [18-20].

Many studies revealed the dental erosion potential of acetic acid, but it was a highly excessive intake of vinegar, and the erosive tooth wear was induced by daily consumption of a glass of apple cider vinegar or more to achieve weight loss [21,22].

Thus, this study aimed to evaluate the effectiveness of apple cider vinegar in dental plaque and gingivitis control in children with CP compared to manual brushing without toothpaste.

## Materials and methods

Study design

This study is a parallel-arm, randomized clinical trial to evaluate the effectiveness of apple vinegar cider and mechanical removal in dental plaque and gingivitis control in children with cerebral palsy. The study was conducted in cerebral palsy centers (Damascus, Syria) between December 2020 and June 2021. The study was approved by the Ethics Committee at the Ministry of Higher Education in Syria (405/SM). All participants' parents were informed of the study's procedure and objectives and were included only after providing informed consent.

Sample size calculation

Sample size calculated was with G* Power 3 (Heinrich-Heine-Universität, Düsseldorf, Germany). The power was set at 95%, with an alpha error probability of 0.05. Therefore, 50 children were in two subgroups, with 25 participants in each.

Participants

Fifty children with cerebral palsy were recruited from cerebral palsy centers, and all participants fulfilled the inclusion criteria: age from three to six years, with primary dentition, have a caregiver to supervise the brushing and ACV applying process, do not suffer from another general systemic disease or seizures, do not take any medication continuously or antibiotics for two weeks before starting the examination and do not have dental abscesses or severe viral infection.

The sample was divided into two subgroups (n=25): group 1 (G1) applying apple cider vinegar 5% on teeth using cotton once a day and for six months; group 2 (G2) performing mechanical brushing without paste once a day for six months.

Intervention

The first examination of the children was done in the presence of the caregiver of each child, and then every participant was given either apple cider vinegar or a toothbrush, according to its group. Caregivers were taught application methods.

Plaque accumulation and gingival status were evaluated using Turesky of the Quigley-Hein plaque index (TQHPI; see Table 1) [23] and modified gingival index (MGI; see Table 2) [24] on buccal surfaces of 55, 61, 64, 75, 81, and 84, according to Ramfjord [25] seven times: pre-intervention baseline (T0), post-intervention at one month (T1), two months (T2), three months (T3), four months (T4), five months (T5), six months (T6).

**Table 1:** A grading system for plaque accumulation

Score	Criteria
0	No plaque present
1	Separate flecks of plaque at the cervical margin
2	A thin continuous back of plaque (up to 1 mm) at the cervical margin
3	A band of plaque wider than 1 mm but covering less than one-third of the side of the crown of the tooth
4	Plaque covering at least one-third but less than two-thirds of the side of the crown of the tooth
5	Plaque covering two-thirds or more of the side of the crown of the tooth

**Table 2:** The grading system of gum status according to modified gingival index (MGI)

Score	Criteria
0	Absence of inflammation
1	Mild inflammation or with slight changes in color and texture but not in all portions of gingival marginal or papillary
2	Mild inflammation, such as the preceding criteria, in all portions of gingival marginal or papillary
3	Moderate, bright surface inflammation, erythema, edema, and/or hypertrophy of gingival marginal or papillary
4	Severe inflammation: erythema, edema, and/or marginal gingival hypertrophy of the unit or spontaneous bleeding, papillary, congestion, or ulceration

Statistical analysis

Statistical analysis was performed using SPSS (IBM, Inc., Armonk, USA). Indices values for continuous valuables were presented as mean and standard deviation. Intragroup comparisons, TQHPI, and MGI scores were analyzed by a two-sample t-test, and inter-observed times were analyzed by paired t-test. A significance level of 0.05 was adopted.

## Results

**Figure 1:**
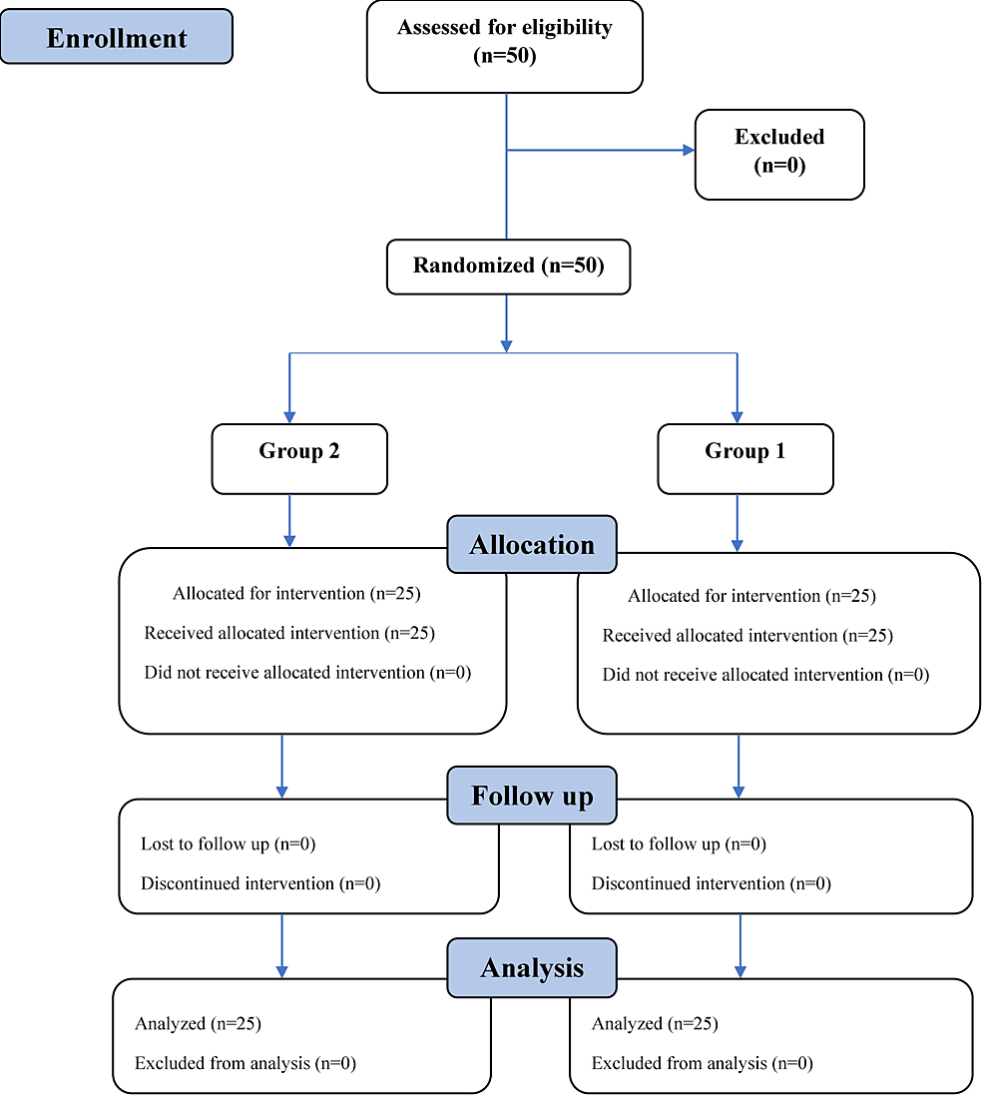
Participants' flow diagram

Participants' inclusion in the study according to the Consolidated Standards of Reporting Trials (CONSORT) is illustrated on the flowchart in Figure 1.

Fifty participants were enrolled in this study, 22 females and 28 males. Means and standard deviations of TQHPI scores are described in Table 3. The manual brushing group showed lower plaque accumulation scores than the ACV group at T1, T2, and T3 (p<0.05). G1 showed lower plaque accumulation scores than G2 (p<0.05) at T5 and T6. At T0 and T4, no significant difference (p>0.05) was observed regarding the amount of plaque accumulation between the G1 and G1. ACV group showed an improvement in plaque accumulation scores in T0 to T6 (p<0.05), except for T3 to T4, which showed no significant difference (p>0.05). The manual brushing group showed an improvement in plaque accumulation score in T0 to T3 (p<0.05), then showed increased dental plaque accumulation in T4 to T6 (p<0.05) except for the two last stages (T5 and T6), which presented no significant difference (p>0.05).

**Table 3:** Mean and standard deviations for each score of plaque accumulation for the TQHPI at the different periods ACV - apple cider vinegar; TQHPI - Turesky of the Quigley-Hein plaque index; G1 - group 1; G2 - group 2 ^a ^two-sample t-test ^b^ paired t-test

Time/groups	Group 1 (ACV)	Group 2 (manual brushing)	p<0.05^a^
T0	1.95±0.47	1.84±0.54	-
T1	1.46±0.55	0.96±0.47	G1>G2
T2	1.03±0.45	0.63±0.36	G1>G2
T3	0.63±0.34	0.38±0.17	G1>G2
T4	0.70±0.36	0.60±0.27	-
T5	0.49±0.28	0.64±0.34	G1
T6	0.40±0.23	0.68±0.39	G1
p<0.05^b^	T0>T1 T1>T2 T2>T3 T4>T5 T5>T6	T0>T1 T1>T2 T2>T3 T3	

Means and standard deviations of MGI scores are described in Table 4. The manual brushing group showed lower MGI scores than the ACV group at T2 (p<0.05). But then G1 showed lower MGI scores than G2 (p<0.05) at T5. At T0, T1, T3, T4, and T6, no significant differences (p>0.05) were observed regarding the gum status between the G1 and G1. Both groups showed an improvement in gum status scores in T0 to T6 (p<0.05), except for T5 to T6, which presented no significant difference (p>0.05) in G1, and all comparisons between T2 and T5 presented no significant difference (p>0.05) in G2.

**Table 4:** Mean and standard deviations for each score of gum status for the (MGI) at the different periods ACV - apple cider vinegar; G1 - group 1; G2 - group 2 ^a^ two-sample t-test ^b^ paired t-test

Time/groups	Group 1 (ACV)	Group 2 (manual brushing)	p<0.05^a^
T0	1.73±0.52	1.36±0.60	-
T1	1.12±0.48	0.99±0.53	-
T2	0.94±0.45	0.55±0.33	G1>G2
T3	0.60±0.16	0.53±0.31	-
T4	0.42±0.20	0.48±0.31	-
T5	0.33±0.17	0.47±0.22	G1
T6	0.40±0.27	0.36±0.18	-
p<0.05^b^	T0>T1 T1>T2 T2>T3 T3>T4 T4>T5	T0>T1 T1>T2 T5>T6	

## Discussion

Daily oral care is a major challenge for young children [26]. These challenges increase in children with special needs, especially those with cerebral palsy, who have a variety of physical and mental conditions that make oral care more difficult, either alone or with a caregiver [27].

The main purpose of this study was to evaluate the effectiveness of methods for reducing oral care difficulties in children with cerebral palsy, therefore, establishing good oral health. This study aimed to evaluate and compare the effectiveness of ACV and mechanical brushing without dentifrices among children with cerebral palsy.

TMQHPI was used to evaluate dental plaque accumulation and MGI to evaluate gingival status on buccal surfaces of modified Ramfjord teeth. This plaque index was chosen because it can detect small differences in the amount of dental plaque, therefore better demonstrating the capacity of plaque removal [23].

This study found that the ACV group showed a significant decrease in plaque accumulation at each time (from T0 to T6), showing that apple cider vinegar dissolves dental plaque. The results reported here are consistent with Salman and Younis, confirming that the use of cider vinegar mouth rinse has reduced the accumulation of dental plaque [20] and agreed with Prasad et al. study that acetic acid could dissolve 25% of dental plaque [18]. Also, Liu and Hannigan found that the five-second use of topical vinegar solved the mature plaque and reduced plaque accumulation [19].

The manual brushing group showed an improvement in plaque accumulation in the first three months (T0 to T3; p<0.05), and that is consistent with many studies that confirmed the effectiveness of mechanical removal in reducing dental plaque accumulation [9,10,28].

Difficult behaviors during brushing, reflex movements, and reflex biting of the dental brush in children with CP can be the reason why toothbrushes wear after three months leading to the lower effectiveness of dental plaque removal. Zhou et al. approved that children with special needs cause excessive toothbrush wear, which is associated with the child's social skills [29], which can explain the following increase in dental plaque in the last three months (p<0.05). Also, many studies found that worn dental brushes (equivalent to three months of use) were less effective in removing dental plaque than completely new ones [30-32]. 

Both groups showed an improvement in gingival status (T0 to T6; p<0.05). As gingival status is associated with dental plaque removal [29], present results showed a decrease in TQHPI scores in both the ACV group and manual brushing group, which leads to a correspondent decrease in MGI; therefore, MGI scores in the manual brushing group decreased at T2 and then increased after T3 [33].

A possible limitation of the study is the partial generalizability of the results to some similar context, which implies a need for further studies of other application methods. Another limitation is the difficulty in finding the required age range for children with cerebral palsy. In addition, it was the parent's responsibility to follow the instructions for a long period (six months).

## Conclusions

To sum up, this in vitro study has demonstrated the possibility of using apple cider vinegar to reduce plaque and gingivitis. In addition, without additives, apple cider vinegar has both mechanical and chemical effects on dental plaque, and it may be a natural, available, inexpensive, and harmless substance that can improve the quality of oral care for difficult groups of children and people with special needs. Unlike toothbrushes, especially electric toothbrushes, they are effective, but they may be expensive and not available to all children.
